# Silver Nanoparticles Biosynthesized from *Metarhizium anisopliae* Crude Extract: Insecticidal Activity, Residual Efficacy, and Metabolic Responses in *Solenopsis invicta*

**DOI:** 10.3390/microorganisms14102334

**Published:** 2026-10-13

**Authors:** Dongxu Wang, Canxia Wu, Wenzhe Li, Huaxin Cai, Jingyang Ni, Yinghua Tong

**Affiliations:** College of Forestry, Fujian Agriculture and Forestry University, Fuzhou 350002, China

**Keywords:** green mycosynthesis, fungal-derived nanomaterials, photostability, biocidal potency, metabolic perturbation

## Abstract

Green biosynthesis of silver nanoparticles using natural biological extracts represents a promising route to develop eco-friendly nanopesticides; nevertheless, many biogenic nanoformulations suffer from poor photostability and rapid loss of bioactivity under field sunlight exposure. In this study, silver nanoparticles (Des-AgNPs) were one-pot biosynthesized using crude fermentation extract from the entomopathogenic fungus *Metarhizium anisopliae* (Metschn.) Sorokīn, which served as both a reducing agent and a surface-capping ligand. *Solenopsis invicta* Buren was employed as a model pest to evaluate the physicochemical features, insecticidal potency, outdoor residual stability, and underlying metabolic toxic mechanism of the resulting nanomaterial. The synthesized Des-AgNPs exhibited high crystallinity, favorable dispersibility, and excellent colloidal stability. Laboratory bioassays demonstrated potent insecticidal activity against *S. invicta* workers, with a 5-day LC_50_ of 174.74 mg/kg. Notably, outdoor exposure assays revealed that Des-AgNPs significantly outperformed the unformulated fungal crude extract in residual efficacy: Des-AgNPs retained high insecticidal activity after 96 h of environmental exposure, whereas the free crude extract underwent a pronounced decline in toxicity within 24 h. Untargeted LC-MS-based metabolomics was further applied to decipher the multi-target toxic responses triggered by Des-AgNPs. In total, 540 differential metabolites (321 upregulated, 183 downregulated) were identified. KEGG pathway enrichment revealed significant disruptions in glutathione metabolism, cytochrome P450-mediated xenobiotic detoxification, tyrosine/dopamine neurotransmitter metabolism, and ABC-transporter-driven efflux pathways. These metabolic perturbations reflected oxidative stress, impaired energy metabolism, compromised cell membrane integrity, disrupted neural transmission, and biomacromolecule damage in the model pest. Collectively, our results demonstrate that entomopathogenic-fungal-extract-capped silver nanoparticles can overcome the photodegradation drawback of free fungal bioactive metabolites. This mycosynthetic strategy provides a feasible framework for constructing stable, high-performance nanobiopesticides, and the workflow could be extended to other fungal extracts and target insect pests beyond the present model system.

## 1. Introduction

Entomopathogenic fungi are important biological control agents because they can infect and kill arthropod pests and produce diverse insecticidal secondary metabolites. *Metarhizium anisopliae* (Metschn.) Sorokīn is a well-studied entomopathogenic fungus whose fermentation-derived crude extract contains bioactive metabolites, including destruxins, cytochalasins, phenolic acids, and peptides, with reported insecticidal activity [1,2]. In addition to their biological activity, compounds in fungal crude extracts can provide hydroxyl, amino, and other functional groups that contribute to the reduction of silver ions and stabilization of newly formed nanoparticles [3]. However, live *M. anisopliae* conidia are known to suffer UV-mediated viability loss under field conditions [4], and crude fungal extracts and their bioactive metabolites may also undergo rapid degradation under environmental exposure, which can limit residual insecticidal efficacy and necessitate repeated application. Therefore, improving the stability and persistence of fungal-extract-derived insecticidal materials is an important objective for their practical use.

Silver nanoparticles (AgNPs) have attracted considerable attention as potential insecticidal materials because of their physicochemical properties and their capacity to induce multiple forms of cellular stress in insects [5]. AgNPs can affect insects through several processes, including silver-ion release, reactive oxygen species accumulation, membrane lipid peroxidation, and interference with essential metabolic enzymes [6]. Compared with conventional physical and chemical approaches, biosynthetic routes for AgNPs can use natural materials as reducing and stabilizing agents and may offer advantages in environmental compatibility, cost, and product stability [7,8]. Plant-derived flavonoids and phenols [9], as well as microbial enzymes, peptides, and other metabolites [10], have been used to reduce silver ions and stabilize nanoparticles. Previous studies have reported insecticidal activity of AgNPs synthesized using plant extracts against several agricultural and forestry pests, including *Coptotermes formosanus* Shiraki [11], *Spodoptera litura* Fabricius [12], and *Aedes aegypti* Linnaeus [13]. In addition, AgNPs synthesized using extracts from entomopathogenic fungi, including *M. anisopliae*, have shown insecticidal activity against *Plutella xylostella* Linnaeus [14]. Together, these findings indicate that biologically synthesized AgNPs may provide a useful platform for developing nanoparticle-based insecticidal formulations.

The red imported fire ant, *Solenopsis invicta* Buren, is an invasive social insect native to South America and is recognized as one of the most destructive invasive pests globally [15,16]. Since its introduction to the United States in the 1930s, *S. invicta* has spread widely in several regions, including Australia, New Zealand, China, and Pacific island nations [17,18]. Its competitive dominance and aggressive stinging behavior can disrupt local ecosystems, reduce agricultural productivity, and pose risks to public health [19]. Current management relies mainly on synthetic insecticides and biological-control preparations, including entomopathogenic fungal products [20]. However, repeated application of chemical insecticides can promote resistance development and environmental contamination [21]. As an economically and ecologically important invasive pest, S. invicta was selected as a model target in this case-study system to evaluate the insecticidal activity and residual efficacy of new biologically derived nanoparticle formulations.

Despite the potential of fungal-extract-mediated AgNPs as insecticidal formulations, evidence remains limited regarding their residual efficacy under outdoor exposure and the physiological pathways associated with their toxicity in target insects. We hypothesized that silver nanoparticles biosynthesized using *M. anisopliae* crude extract would retain insecticidal activity for longer than the crude-extract formulation under the tested outdoor exposure conditions and would induce measurable metabolic perturbations in *S. invicta* workers. Building upon this hypothesis, in the present study, biosilver nanoparticles were synthesized using crude extract from the entomopathogenic fungus *M. anisopliae*. We selected *S. invicta* as an insect model to evaluate the insecticidal activity, residual efficacy, and metabolic perturbations induced by these biosynthesized silver nanoparticles. This work aims to provide insights into the biocidal potential of fungus-derived silver nanoparticles for insect pest control.

Despite the potential of fungal-extract-mediated AgNPs as insecticidal formulations, evidence remains limited regarding their residual efficacy under outdoor exposure and the physiological pathways associated with their toxicity in target insects. We hypothesized that silver nanoparticles biosynthesized using *M. anisopliae* crude extract would retain insecticidal activity for longer than the crude-extract formulation under the tested outdoor exposure conditions and would induce measurable metabolic perturbations in *S. invicta* workers. Using *M. anisopliae* and *S. invicta* as a case-study model system, this study aims to test the above-mentioned hypothesis and gain mechanistic insights into the biocidal potential of fungus-derived silver nanoparticles, supporting future development of stable nanobiopesticides for insect pest management.

## 2. Materials and Methods

### 2.1. Test Insects and Metarhizium anisopliae Strain

The test *S. invicta* were collected in a single batch from Fujian Agriculture and Forestry University (26°08′04.7″ N, 119°13′07.1″ E), with enough individuals reserved to complete all subsequent experiments. After collection, the ant colony was transferred to a plastic feeding box (40 cm × 30 cm × 15 cm) lined with talcum powder on the inner wall and reared indoors for a week. During the acclimation period, unhealthy, injured or pathogen-infected worker ants were eliminated daily to avoid background mortality caused by pre-existing fungal or bacterial infection. The ants were continuously fed with 10% (*w*/*v*) sucrose solution.

The test strain, *M*. *anisopliae* Ma1775, was isolated from larvae of *Monochamus alternatus* Hope collected in Tsukuba, Ibaraki Prefecture, Japan. It was kindly provided by the Institute of Forest Protection, Fujian Academy of Forestry and preserved by the Forest Protection Teaching and Research Section of Fujian Agriculture and Forestry University [22].

### 2.2. Test Solutions

Silver nitrate (99.8%, Sinopharm Group, Beijing, China), ethyl acetate (analytical grade, Xilong Science, Shantou, China), dichloromethane (analytical grade, Xilong Science, Shantou, China), methanol (LC-MS grade, Merck, Darmstadt, Germany), acetonitrile (LC-MS grade, Shanghai Xingke, Shanghai, China), and formic acid (LC-MS grade, Aladdin, Shanghai, China) were used.

### 2.3. Extraction of Metarhizium anisopliae Crude Extract

The test strain was *M*. *anisopliae* Ma1775, a laboratory-preserved strain. After reviving the Ma1775 strain three times using *S. invicta* workers, it was inoculated onto PDA solid medium (200 g potato, 20 g glucose, 10 g peptone, 20 g agar, pH left unadjusted) plates and incubated at a constant temperature of (26 ± 1) °C until spore production was complete. After revitalization, a spore suspension at a concentration of (1.0 ± 0.2) × 10^7^ spores/mL was inoculated into 200 mL of SDAY liquid medium (10 g yeast extract, 40 g glucose) and fermented in a shaking water bath (26 °C, 210 rpm) for 9 days.

The crude extract was prepared according to the method of Hu et al. [22]. The fermentation broth was filtered, and the filtrate was adjusted to a pH of 3.0 after centrifugation to remove solids. It was then extracted three times using a mixed solvent of ethyl acetate and dichloromethane (1:1, *v*/*v*). The organic phases were combined and rotary-evaporated under reduced pressure at 60 °C to remove most of the organic solvent, yielding an oily paste before subsequent oven-drying. Finally, the mixture was dried at a constant temperature of 60 °C to obtain the crude extract of *M. anisopliae*. The resulting crude extract was subsequently used for the biosynthesis of AgNPs.

### 2.4. Biosynthesis and Characterization of Des-AgNPs

#### 2.4.1. Synthesis of Des-AgNPs

To obtain an aqueous solution of the crude extract, 1 g of crude extract from *M. anisopliae* was weighed, mixed with 10 mL of distilled water, and mixed thoroughly. This solution was then slowly added dropwise to 90 mL of a 5 mmol/L AgNO_3_ solution under continuous stirring to ensure uniform mixing. Subsequently, the pH of the mixture was adjusted to 6 using hydrochloric acid and sodium hydroxide solutions. After thorough stirring, the mixture was placed in a constant-temperature water bath (80 °C) and heated for 80 min, with shaking performed every 20 min. After the reaction, the mixture was removed and allowed to cool naturally to room temperature. The reaction mixture was then centrifuged at 10,000 rpm for 15 min, and the supernatant was discarded. The resulting precipitate was washed by adding an appropriate amount of distilled water and centrifuged again, a process that was repeated three times. Finally, the black precipitate was air-dried for 4 h to obtain the powder of *M. anisopliae* crude extract silver nanoparticles (Des-AgNPs), which was then sealed and stored at 4 °C until further use.

#### 2.4.2. Characterization of AgNPs

Des-AgNPs were characterized using ultraviolet–visible (UV-Vis) spectroscopy, transmission electron microscopy (TEM), X-ray diffraction (XRD), and dynamic light scattering (DLS). UV-Vis, TEM, XRD and DLS potential characterizations were performed by an independent commercial analytical testing service, with all key measurement parameters provided below. For UV-Vis measurements, 1 mg of Des-AgNPs powder was dispersed in 2 mL of distilled water. The sample solution was prepared by dispersing the mixture uniformly using a constant-temperature metal mixer. Scans were performed on a UV-2600 UV-Vis spectrophotometer that had been preheated for 20 min and baseline-corrected. Each sample was tested three times, and the data were analyzed using Origin 2022 software. For TEM observation, Des-AgNPs were dispersed in anhydrous ethanol by ultrasonication. A drop of the suspension was applied to a copper mesh support film; after standing for 5 min, excess liquid was removed, and the sample was dried at room temperature before observation and image acquisition. For XRD analysis, 0.3 g of Des-AgNPs powder was ground and loaded into the sample holder. Measurements were performed at room temperature using a Cu target (*λ* = 1.5406 Å) under conditions of 40 kV and 40 mA, with a scanning range of 2θ = 10–90° at a rate of 4°/min, and the diffraction patterns were recorded. For DLS and zeta potential measurements, 20 mg of Des-AgNPs was weighed and dispersed in 2 mL of distilled water. After mixing, the solution was injected into the sample cell, and the hydrodynamic particle size and zeta potential were measured at 25 °C using a Zetasizer ZS90 preheated for 30 min; each sample was measured three times, with three measurement cycles per run.

### 2.5. In Vitro Toxicity Assay of Des-AgNPs on Solenopsis invicta Workers

Following a modified method by Neff et al. [23], a base bait was prepared by mixing 50 g of corn flour, 30 g of wheat flour, 6 g of fish meal, and 4 g of peanut oil, with a small amount of distilled water added and stirred to form pellets with a diameter of 1.2–1.5 mm. Des-AgNPs powder was dissolved in acetone and added to the base bait in small, uniform increments using a pipette, resulting in five bait formulations with AgNPs concentrations (mg/kg) of 1000, 500, 250, 100, and 50. A base bait without AgNPs served as the blank control. An AgNO_3_ treatment was included as an additional control at a concentration equivalent to that used for the preparation of Des-AgNPs. The control bait was treated with an equivalent volume of acetone.

The toxicity was determined using the bait method. *S. invicta* workers were transferred to transparent food trays (16 cm × 11 cm × 6 cm) with talcum powder applied to the inner walls. Sterile water-soaked cotton wool was placed inside the trays to maintain humidity. The trays were placed in a climate chamber maintained at 25 ± 1 °C and 80 ± 5% relative humidity for rearing. A 5-g Des-AgNPs bait was placed in the center of each tray. Each Des-AgNPs concentration gradient constituted one treatment, with five replicates per treatment, each containing more than 50 worker ants. Mortality observations were performed daily at 15:00, ants were considered dead if they showed no response to light touch. Observations continued until all worker ants in one of the treatments had died, at which point the mortality rate was calculated. In this study, all worker ants in one treatment group were dead on day 9 of the experiment, so the observation period was set to 9 days.

### 2.6. Assessment of the Virulence Stability of Des-AgNPs

On sunny days with outdoor temperatures ranging from 28–35 °C and relative humidity of 70–90%, one unified batch of 1000 mg/kg Des-AgNP bait and an equivalent batch of pure *M. anisopliae* crude extract bait were used to minimize experimental variables. Aliquots from the same batch were separately exposed outdoors for 24, 48, 72, 96, and 120 h, respectively. After each scheduled exposure duration, the corresponding bait subsample was collected, and its insecticidal virulence against *S. invicta* workers was tested following the identical bioassay procedure described in Section 2.5. Under the tested preparation and exposure conditions, the results support the conclusion that the Des-AgNP formulation retained its insecticidal activity for longer than the crude-extract formulation.

### 2.7. Assessment of the Effects of Des-AgNPs on the Metabolism of Solenopsis invicta Workers

#### 2.7.1. Sampling

Healthy *S. invicta* workers were selected and fed Des-AgNPs bait for 3 days; 50 mg of live ants were then collected and rapidly snap-frozen in liquid nitrogen and stored at −80 °C. A group fed bait without AgNPs served as the control, with five replicates per treatment.

#### 2.7.2. Metabolite Extraction and LC-MS Analysis

The frozen insect specimens were thawed, chopped into small pieces, and mixed thoroughly. A 20 mg portion was weighed out and placed into a centrifuge tube. The sample was homogenized in a ball mill at 30 Hz for 20 s, followed by centrifugation at 3000 rpm and 4 °C for 30 s. Then, 400 μL of 70% methanol–water extraction solution containing internal standard was added to the pellet; the mixture was shaken at 1500 rpm for 5 min, incubated on ice for 15 min, and centrifuged at 4 °C and 12,000 rpm for 4 min. A 200 μL aliquot of the supernatant was collected for subsequent analysis.

Analysis was performed using ultra-high-performance liquid chromatography–mass spectrometry (UPLC-MS). A Waters ACQUITY Premier HSS T3 Column (1.8 µm, 2.1 mm × 100 mm) was adopted, with a column temperature of 40 °C, a flow rate of 0.4 mL/min, and an injection volume of 4 μL. The mobile phase was 0.1% formic acid in water for positive ion mode and 0.1% formic acid in acetonitrile for negative ion mode.

### 2.8. Statistical Analysis of Data

SPSS 26.0 software was used to analyze the toxicity data. Probit regression analysis was employed to calculate the median lethal time (LT_50_), 5-day median lethal concentration (LC_50_), 95% confidence interval, toxicity regression equation, and coefficient of determination (R^2^) for the Des-AgNPs treatment group. Plots were generated using Origin 2022 software. After preprocessing, the metabolomics data were imported into SIMCA-P 14.1 for principal component analysis (PCA) [24], orthogonal partial least squares discriminant analysis (OPLS-DA) [25], volcano plot analysis [26,27], clustering heatmap analysis [28,29], and KEGG enrichment analysis [30]. The calculation formulas are as follows:

Cumulative mortality rate/% = Number of dead insects/Total number of test insects × 100%(1)

(2) $$\begin{array}{l} \mathrm{Cumulative}\;\mathrm{adjusted}\;\mathrm{mortality}\;\mathrm{rate}/\%\;=\;(\mathrm{Mortality}\;\mathrm{rate}\;\mathrm{of}\;\mathrm{treatment}\;\mathrm{group}-\\ \mathrm{Mortality}\;\mathrm{rate}\;\mathrm{of}\;\mathrm{control}\;\mathrm{group})/(1-\mathrm{Mortality}\;\mathrm{rate}\;\mathrm{of}\;\mathrm{control}\;\mathrm{group})\times 100\% \end{array}$$

## 3. Results

### 3.1. Characterization of Biosynthesized Des-AgNPs

**Figure 1:**
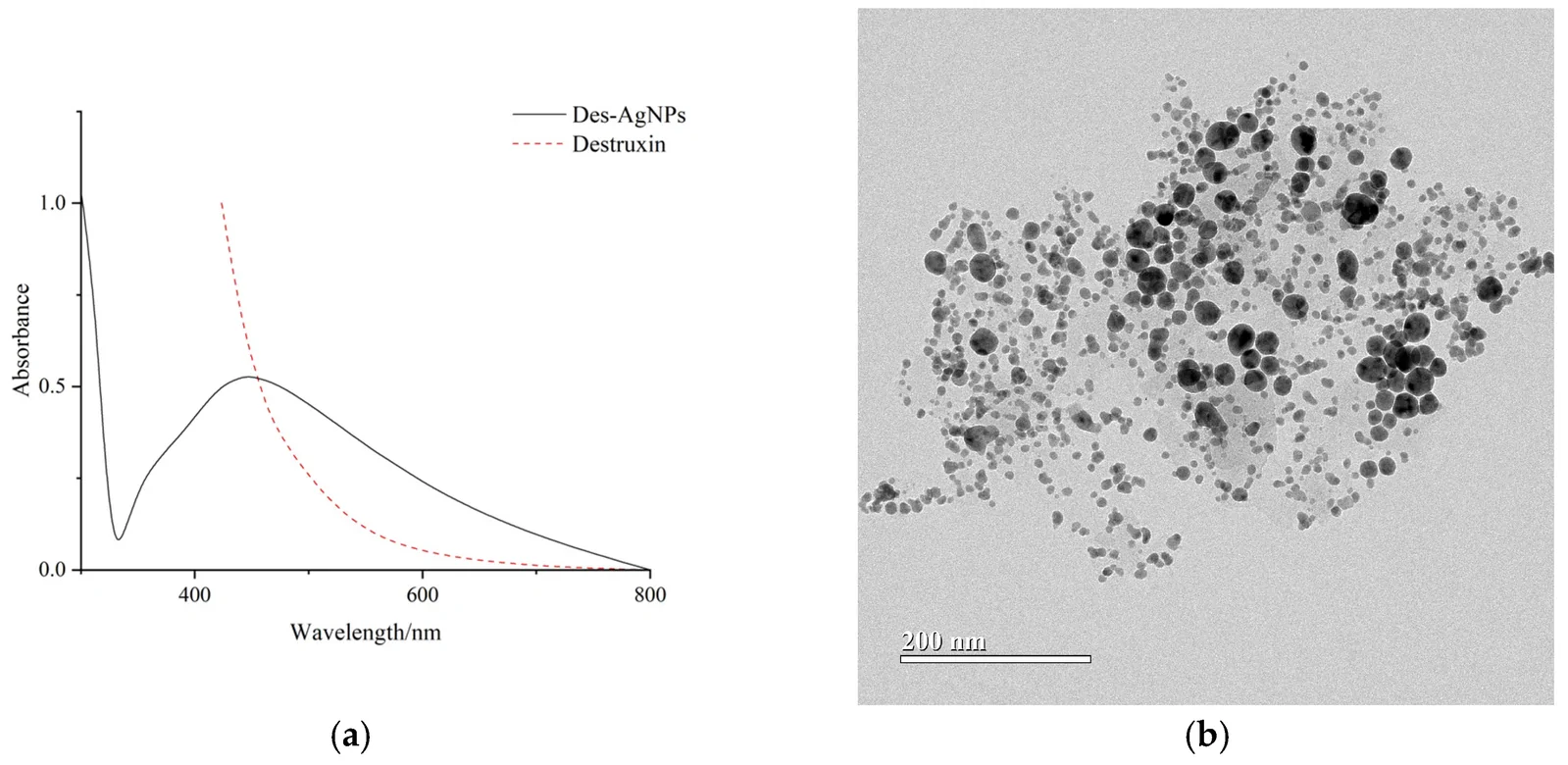

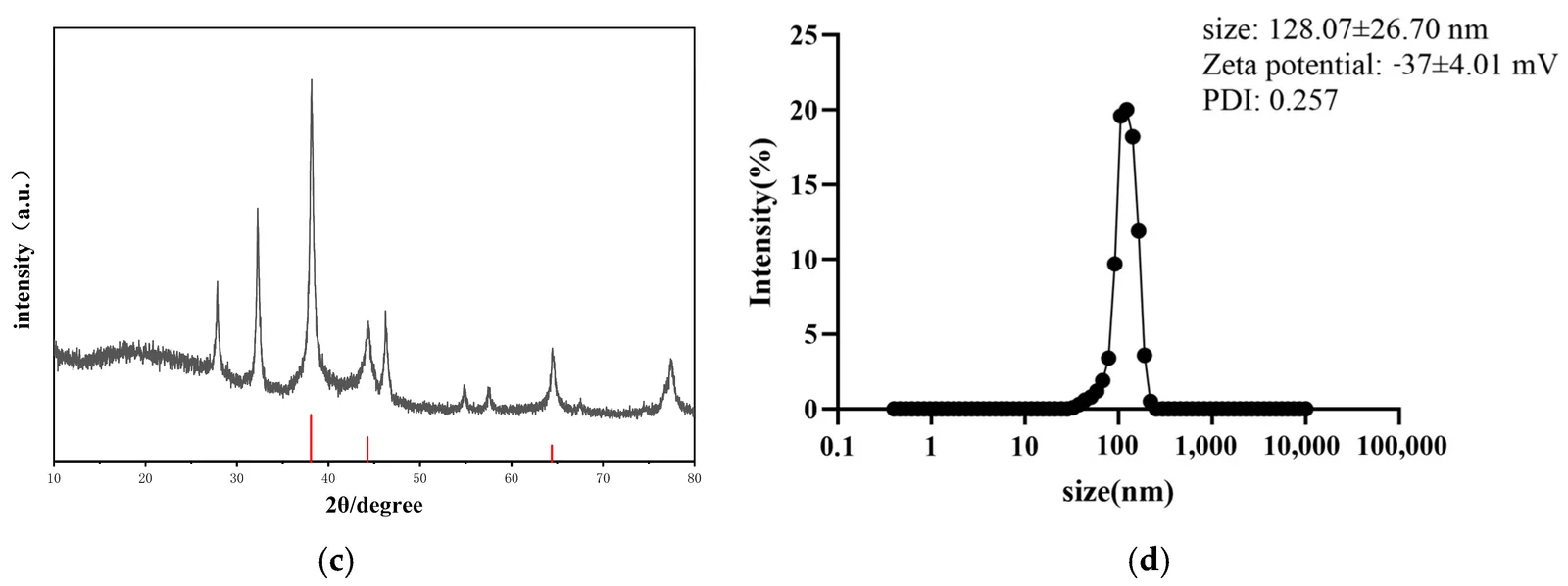
Characterization of biosynthesized silver nanoparticles (Des-AgNPs). (**a**) UV-Vis absorption spectrum; (**b**) TEM image; (**c**) XRD pattern; (**d**) DLS plot.

UV-Vis spectroscopy results (Figure 1a) showed that the *M*. *anisopliae* crude extract solution exhibited no characteristic absorption peaks in the 300–800 nm range, whereas the Des-AgNPs colloidal solution displayed typical surface plasmon resonance absorption peaks at 430–460 nm, with a maximum absorption wavelength of 458 nm. TEM images (Figure 1b) showed that Des-AgNPs were primarily spherical or nearly spherical, with rounded edges and relatively uniform dispersion; particle size statistics (*n* = 100) indicated an average particle size of 49.94 nm. The XRD pattern (Figure 1c) exhibited distinct diffraction peaks near 2θ = 38°, 44°, 64°, and 77°, corresponding to the (111), (200), (220), and (311) crystal planes of the face-centered cubic structure of metallic silver, consistent with entry 87-0717 in the JCPDS database, indicating good crystallinity of the product. DLS (Figure 1d) measurements indicated that the average hydrodynamic particle size of Des-AgNPs was 128.07 nm; the zeta potential was −37.4 mV, with an absolute value greater than 30 mV, indicating that these nanoparticles exhibited good dispersion and colloidal stability in the aqueous dispersion system.

### 3.2. The Toxicity of Des-AgNPs to Solenopsis invicta Workers

**Figure 2:**
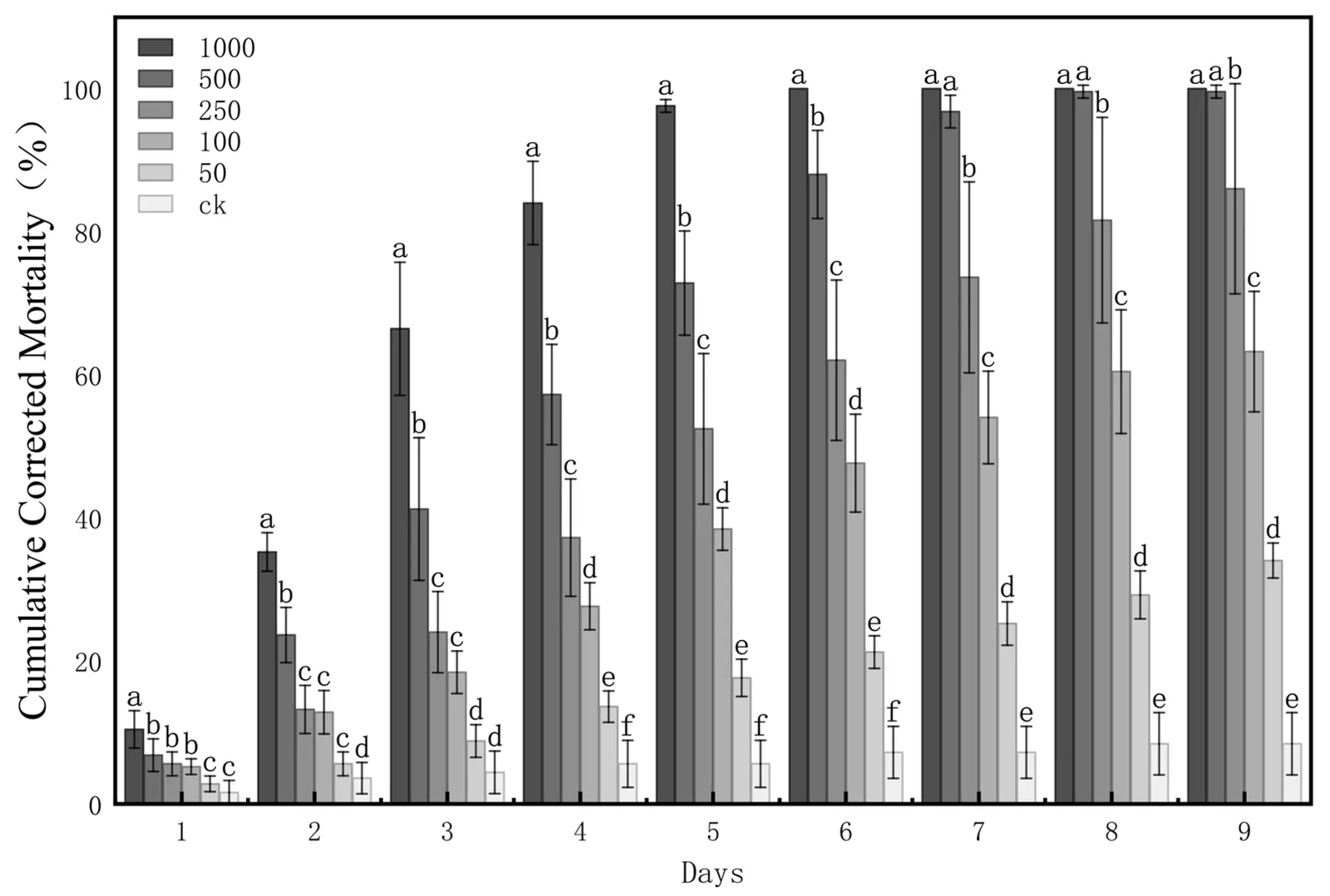
Cumulative corrected mortality of *Solenopsis invicta* workers exposed to five concentrations of Des-AgNPs for 9 days. Note: Different lowercase letters above the bars indicate significant differences among different treatments at the same time point (*p* < 0.05).

Full mortality of fire ant workers was first observed in one treatment group on Day 9 of the bioassay, rendering 9 days the maximum observation period for this trial. As shown in Figure 2, the cumulative mortality rates in all treatment groups at each time point were significantly higher than those in the control group (CK) (*p* < 0.05). An analysis of the toxicity of Des-AgNPs to *S. invicta* is presented in Table 1. For the five concentration gradient treatments, the cumulative adjusted mortality rates of worker ants were all significantly higher than those of the control (CK), with the 1000 mg/kg and 500 mg/kg treatment groups both reaching a cumulative adjusted mortality rate of 100% by day 9. Calculated using the toxicity regression equation, the 5-day LC_50_ of Des-AgNPs for *S. invicta* workers was 174.74 mg/kg.

### 3.3. Analysis of the Stability of Des-AgNPs’ Toxicity to Solenopsis invicta

The toxicity of Des-AgNPs to *S. invicta* workers following bait exposure was analyzed, and the results are shown in Table 2. As shown in Table 2, 24 h after exposure to *M. anisopliae* crude extract, the adjusted mortality rate of *S. invicta* workers began to decrease significantly (*p* < 0.05), and the LT50 increased. In contrast, the cumulative adjusted mortality rate for Des-AgNPs remained at 95.00% even after 96 h of exposure, and the toxicity did not begin to decrease significantly until 120 h, indicating that Des-AgNPs retained insecticidal activity for a longer period under the tested exposure conditions.

### 3.4. Metabolomic Analysis of Solenopsis invicta Workers Following Treatment with Des-AgNPs

#### 3.4.1. Principal Component Analysis and Orthogonal Partial Least Squares Discriminant Analysis

**Figure 3:**
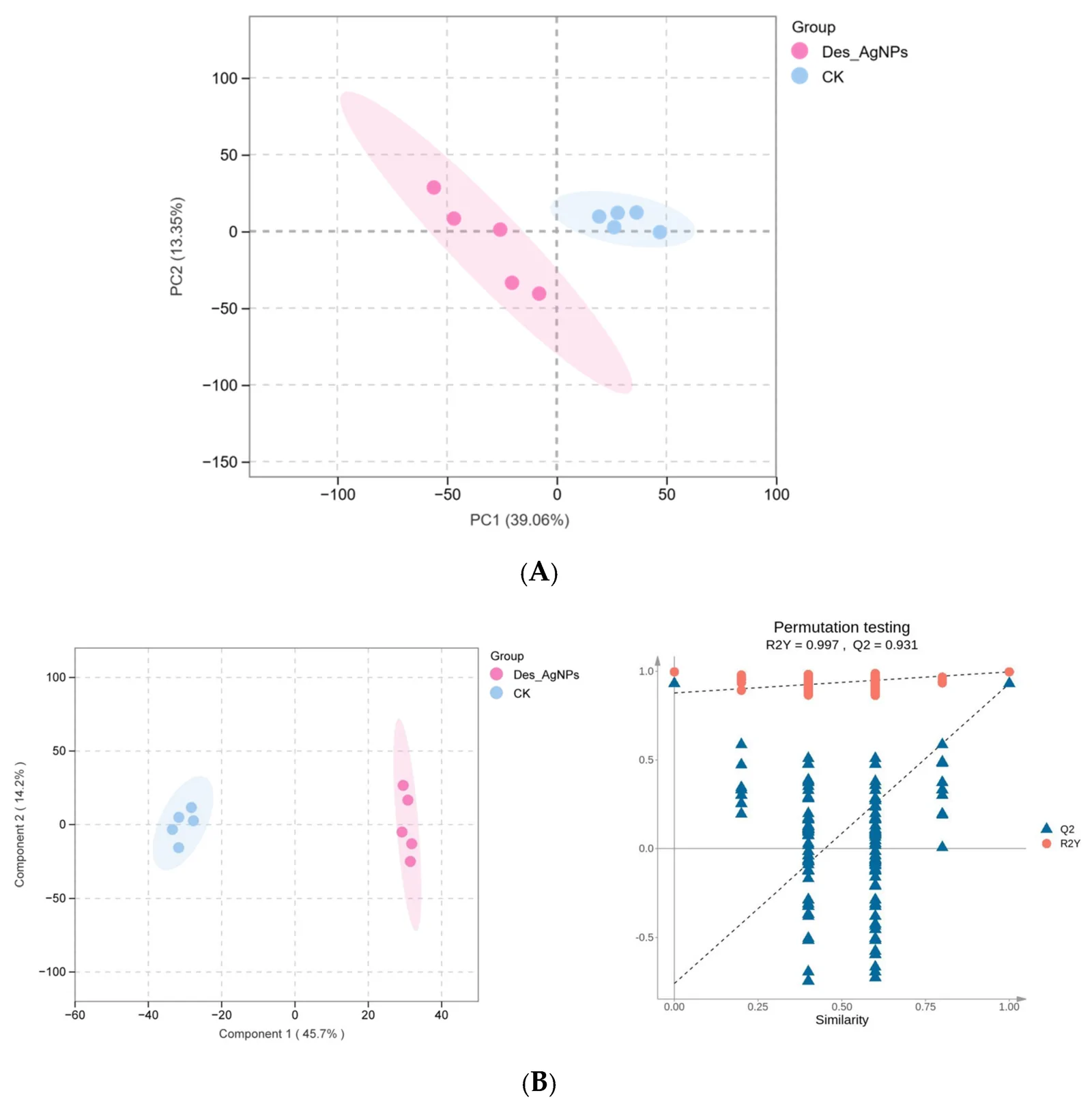
(**A**) Principal component analysis (PCA) of *Solenopsis invicta* workers treated with Des-AgNPs. (**B**) OPLS and OPLS-DA plots of biosynthesized silver nanoparticles.

The results of principal component analysis (PCA) (Figure 3A) showed that the metabolic profiles of *S. invicta* workers in the Des-AgNPs treatment group and the blank control group were clearly separated along the first principal component (contribution rate: 39.06%), with the control group samples showing good clustering and the treatment group samples exhibiting a dispersed distribution. This indicates that Des-AgNPs stress significantly altered the metabolism of *S. invicta* workers, and that there were certain differences in the metabolic responses of individual ants within the colony to this stress. Orthogonal Partial Least Squares Discriminant Analysis (OPLS-DA) (Figure 3B) showed that samples from the treatment group and the control group were distributed in distinct regions on the score plot, with no overlap in the 95% confidence intervals. The model’s Q^2^ value was greater than 0.931, and R^2^Y was 0.997. The model’s robustness was validated via 200 permutation tests, confirming no overfitting. It can be effectively used for subsequent screening and analysis of differential metabolites, confirming that Des-AgNPs exert a significant and targeted metabolic disruption on *S. invicta* workers.

#### 3.4.2. Screening of Differential Metabolites

**Figure 4:**
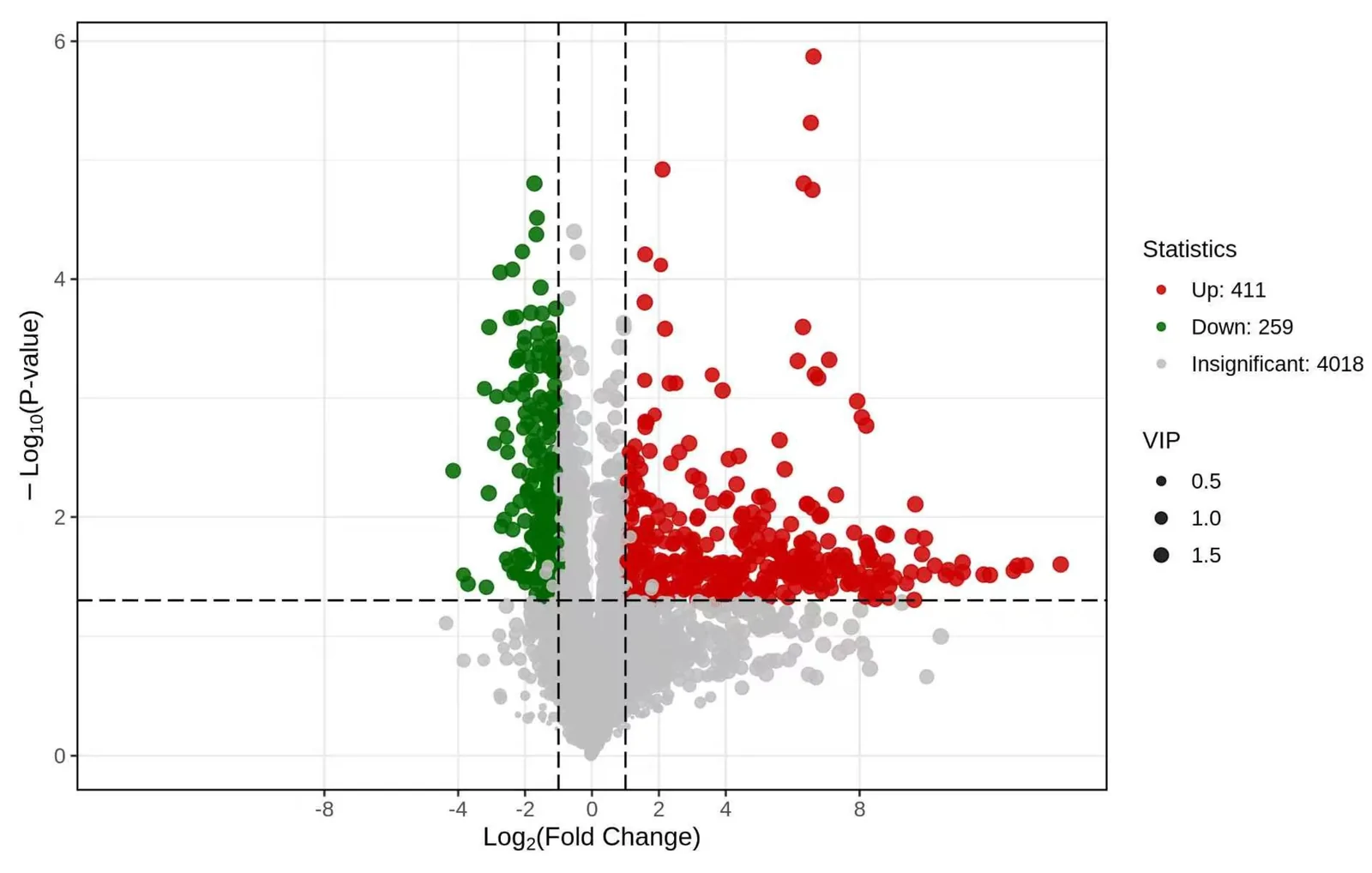
Volcano plot of differential metabolites in *Solenopsis invicta* workers treated with Des-AgNPs.

Using the screening criteria of VIP > 1, *p* < 0.05, and |log_2_ FC| ≥ 2, a differential analysis was performed on metabolites between the Des-AgNPs-treated group and the control group. A total of 540 differential metabolites were identified. After excluding exogenous contaminants, a volcano plot was generated (Figure 4), showing that 321 metabolites were significantly upregulated and 183 were significantly downregulated, while the remaining metabolites did not meet the directionality classification criteria. The results indicate that the metabolic profile of *S. invicta* workers underwent significant changes following Des-AgNPs treatment, with the number of upregulated metabolites exceeding that of downregulated metabolites.

#### 3.4.3. Hierarchical Clustering Analysis of Differential Metabolites

**Figure 5:**
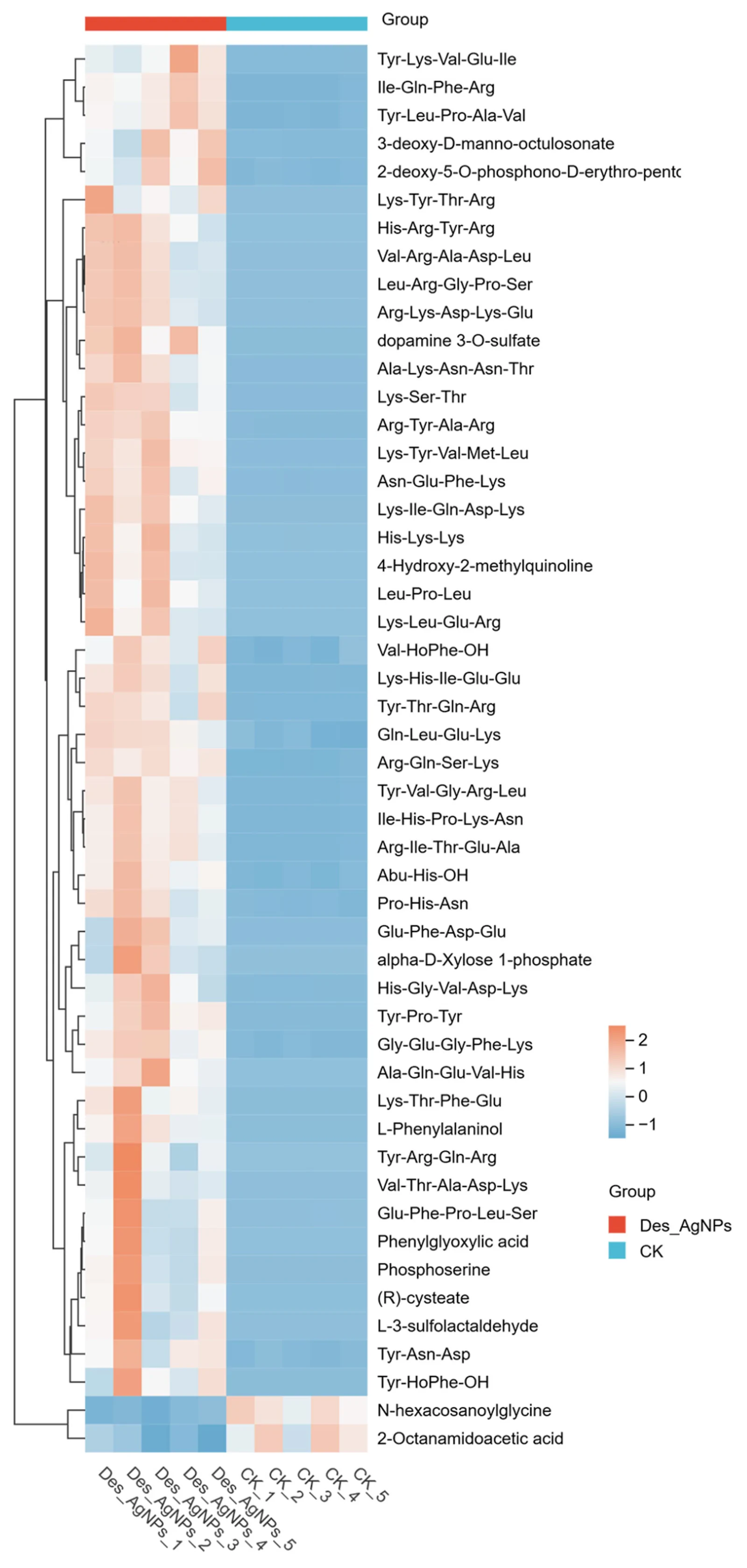
Cluster heatmap of the top 50 differential metabolites in *Solenopsis invicta* workers treated with Des-AgNPs.

The top 50 differential metabolites in the Des-AgNPs-treated group were selected in descending order based on their VIP values and subjected to hierarchical clustering analysis (Figure 5). The results showed significant intergroup differences and intragroup similarities in the metabolic expression patterns between the treated and control groups, indicating that the metabolic profiles of *S. invicta* workers underwent significant changes following treatment. Following Des-AgNPs treatment, dopamine-3-O-sulfate, 2-deoxy-5-O-phosphorylated D-erythritol-pentose, short peptides, and lysophospholipids were significantly upregulated, while structural phospholipids and fatty acid metabolic intermediates were significantly downregulated. These metabolites are primarily involved in neurotransmitter metabolism, nucleic acid-related metabolism, energy metabolism, protein homeostasis, and cell membrane integrity in *S. invicta* workers.

#### 3.4.4. Analysis of Enriched Pathways for Differential Metabolites

**Figure 6:**
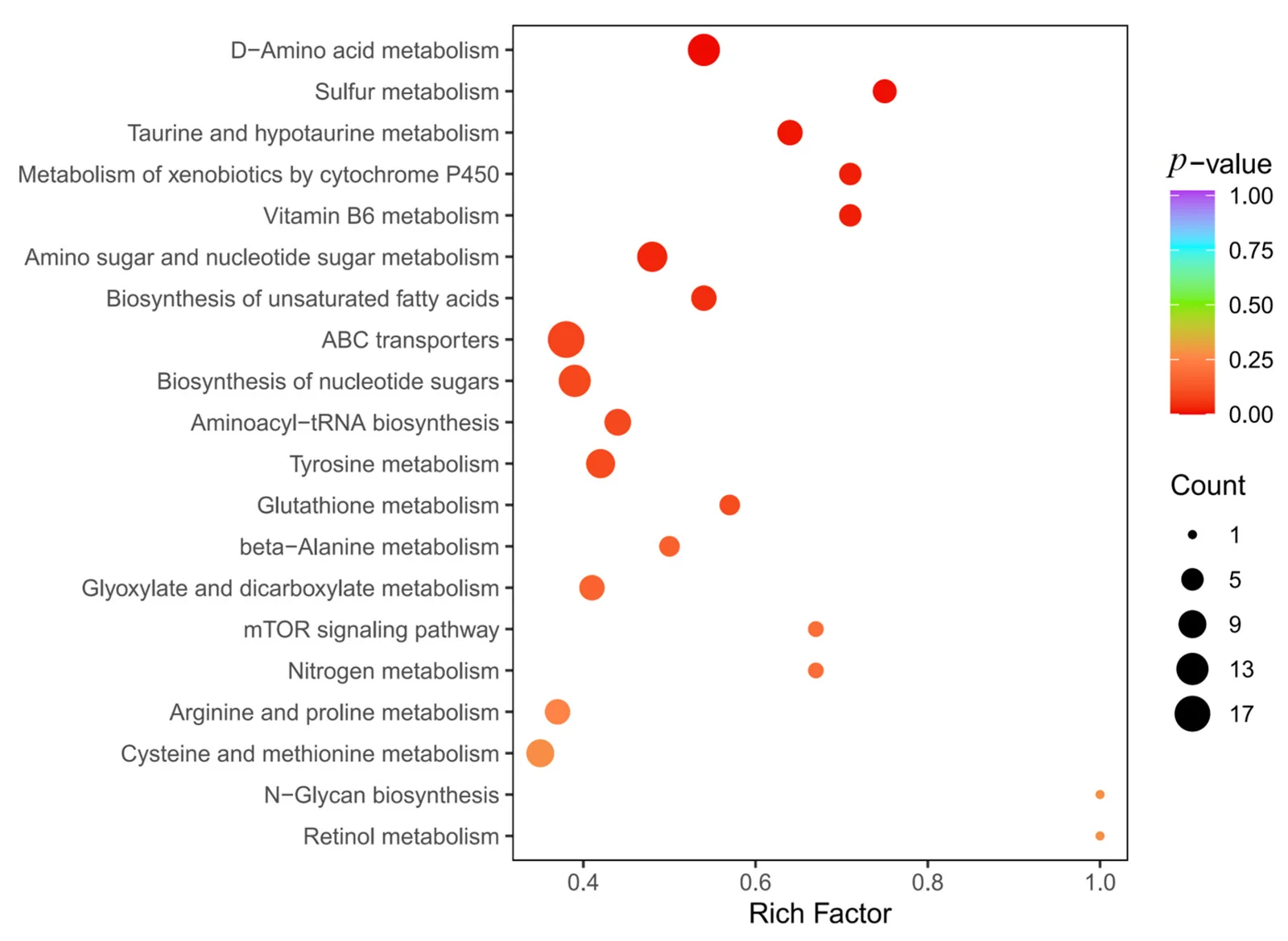
KEGG enrichment analysis of differential metabolites in *Solenopsis invicta* workers treated with Des-AgNPs.

KEGG enrichment analysis (Figure 6) revealed that, following treatment with Des-AgNPs, differential metabolites in *S. Invicta* workers were significantly enriched in the following pathways: Metabolism of xenobiotics by cytochrome P450, glutathione metabolism, sulfur metabolism, tyrosine metabolism, amino sugar and nucleotide sugar metabolism, and ABC transporters (*p* < 0.05). The results indicate that the differentially expressed metabolic pathways in *S. invicta* workers following Des-AgNPs treatment primarily involve the antioxidant and detoxification systems, neurotransmitter metabolism, transmembrane toxin efflux systems, and overall energy and substrate metabolism.

## 4. Discussion

In this study, *M*. *anisopliae* crude extract was used as a reducing agent and stabilizer to biosynthesize Des-AgNPs. UV-Vis spectroscopy revealed that the product exhibited typical surface plasmon resonance absorption peaks consistent with those of AgNPs synthesized by Kumar et al. [31]. TEM and XRD characterization revealed that the AgNPs were spherical or nearly spherical with high crystallinity, and the product exhibited characteristic diffraction peaks of face-centered cubic silver. These diffraction peaks may be attributed to the stabilization of the nanoparticles by *Metarhizium* crude extract acting as a capping agent [15,32]. DLS and zeta potential results confirmed that Des-AgNPs exhibited a narrow particle size distribution and strong electrostatic stability in an aqueous dispersion system, making them resistant to agglomeration; their physicochemical properties meet the application requirements for nanopesticides [33].

Results from in vitro toxicity and toxicity stability assays indicated that Des-AgNPs exhibited strong toxicity against *S. invicta* workers. Compared to the crude extract of *M*. *anisopliae*, Des-AgNPs demonstrated greater toxicity stability and residual efficacy following exposure. Prasad et al. confirmed that AgNPs as a carrier can protect bioactive components, enhance resistance to photodegradation and environmental degradation, and significantly extend residual efficacy [34]. This confirms that nanostructures can effectively address the issues of easy degradation and short residual efficacy associated with traditional bio-pesticides, demonstrating significant potential for field application.

Metabolomic analysis revealed that Des-AgNPs disrupted the overall metabolism of *S. invicta* workers. Differential metabolites were enriched in key pathways such as detoxification, antioxidant defense, neurotransmitter metabolism, and transmembrane transport, providing preliminary insights into the metabolic responses associated with Des-AgNPs exposure and oxidative stress response patterns of AgNPs in *S. invicta* workers.

Des-AgNPs induced oxidative stress in *S. invicta* and activated systemic detoxification and antioxidant responses. KEGG enrichment analysis showed that glutathione metabolism, sulfur metabolism, and the cytochrome P450 pathway were highly enriched. This suggests that when subjected to AgNP stress, *S. invicta* workers redirect a large number of sulfur-containing precursors toward the synthesis of antioxidant molecules such as glutathione to scavenge excess reactive oxygen species (ROS). This aligns with the findings reported by Wu et al. [35], who concluded that AgNP-induced ROS bursts trigger oxidative stress, leading to the depletion of the antioxidant system and resulting in damage to biomacromolecules and cell membranes. Once biosynthesized AgNPs enter the insect’s body, they continuously release Ag^+^, inducing a ROS burst that causes oxidative damage to cell membranes, proteins, and nucleic acids [36,37]. To counteract this stress, *S. invicta* workers enhance sulfur metabolism flux, accelerating the conversion and utilization of sulfur-containing amino acids such as cysteine and methionine to maintain the glutathione cycle and reductive homeostasis. Concurrently, the cytochrome P450 pathway is significantly activated, reflecting enhanced recognition, binding, and metabolic detoxification of exogenous nanoparticles—a typical defense mechanism by which insects respond to xenobiotic stress [38,39]. However, as exposure time increases and doses accumulate, the antioxidant system is gradually depleted, oxidative damage becomes irreversible, and ultimately leads to loss of cellular function.

Furthermore, Des-AgNPs disrupt the structural integrity of the cell membrane, leading to disturbances in membrane lipid metabolism and the leakage of intracellular contents. Metabolomic data showing an upregulation of lysophospholipids and a downregulation of structural phospholipids indicate that Des-AgNPs can cause damage to the cell membrane structure and increased permeability by activating phospholipase A2 or inducing lipid peroxidation [40,41,42,43]. Cell membrane damage induces mitochondrial rupture and impaired energy metabolism, leading to cell death. In this study, the widespread downregulation of fatty acid metabolic intermediates directly demonstrates that mitochondrial fatty acid β-oxidation is inhibited, ATP synthesis is drastically reduced, and *S. invicta* die rapidly due to energy depletion [44,45].

At the same time, Des-AgNPs interfere with genetic information transmission and protein function. The significant upregulation of 2-deoxy-5-O-phosphate-D-erythrobios in the metabolome suggests abnormalities in DNA synthesis and repair processes, indicating that the body attempts to initiate damage repair but is unable to fully reverse the damage [40]. Studies have shown that AgNPs can disrupt DNA strand structure by releasing Ag^+^ ions that bind to phosphorus [39,46], while simultaneously binding to sulfhydryl groups in proteins, leading to enzyme denaturation [43,47]. The enrichment of a large number of short peptides demonstrates widespread protein degradation and abnormal hydrolysis within the body, resulting in the collapse of protein homeostasis and further exacerbating cell death and tissue necrosis [44].

In addition to membrane structural and metabolic damage, Des-AgNPs significantly disrupt neurotransmitter metabolism in *S. invicta*. The upregulation of dopamine-3-O-sulfate among differential metabolites suggests that dopaminergic signaling is inactivated by excessive sulfation, thereby impairing neural transmission [48,49]. Significant enrichment of tyrosine metabolic pathways confirms disruptions in the synthesis, transport, and degradation of catecholamine neurotransmitters [50]. For social insects such as *S. invicta*, abnormal neural transmission directly leads to disorientation, anorexia, and the disruption of nest-guarding and mutual feeding behaviors in worker ants, thereby effectively suppressing the entire colony [27,40]. AgNPs interfere with the nervous system primarily by inhibiting key enzymes such as acetylcholinesterase, disrupting synaptic membrane structures, and blocking ion channels, ultimately leading to paralysis and death [39,49].

Ultimately, Des-AgNPs can induce the activation of the ABC transporter pathway in *S. invicta*. The insects actively transport exogenous toxins in and out of the body by activating the transmembrane efflux system, thereby reducing the accumulation of toxins within the body. This represents a typical stress-tolerant defense mechanism in insects against nanotoxic stress [35,38]. However, the membrane-penetrating and sustained-release capabilities of Des-AgNPs allow them to continuously bypass *S. invicta*’s detoxification defense system, making it difficult for the efflux function of ABC transporters to counteract toxic damage. Therefore, the activation of this pathway can only delay the poisoning process and cannot fundamentally reverse the lethal effects caused by oxidative stress, membrane damage, and metabolic disorders [35,39].

In summary, the toxic effects of Des-AgNPs on *S. invicta* workers result from the synergistic interaction of multiple pathways, including oxidative stress bursts, disruption of cell membrane structure, energy metabolism failure, damage to biomolecules, and inhibition of neural transmission. Des-AgNPs exert a multi-level assault ranging from cellular structure and energy metabolism to genetic material and neural signaling, ultimately triggering systemic metabolic collapse and rapid mortality of ants. Although direct chemical characterization of the fungal crude extract was not performed in this work, previous studies have demonstrated that the fermentation broth and culture filtrates of *M*. *anisopliae* contain a range of bioactive secondary metabolites, including the well-characterized insecticidal destruxins A, B, and E, which can be recovered from fermentation-derived extracts [51,52,53,54]. Based on this established consensus, we infer that the toxic activity of the crude extract in this study is mainly derived from such secondary metabolites, and the synergistic effect between these active components and silver nanoparticles contributes to the enhanced insecticidal efficiency.

The present research has several inherent limitations that should be acknowledged. First, although previous studies have reported that the fermentation crude extract of *M*. *anisopliae* contains major insecticidal metabolites including destruxins and cytochalasins, targeted chemical identification and quantitative detection of bioactive components in the crude extract were not performed in this work. Systematic identification of the specific components in our crude extract via LC-MS will be carried out in our follow-up research to verify this inference. Second, the indoor lethal toxicity bioassays lacked an independent treatment group solely treated with fungal crude extract as the positive control. In addition, because the fungal biomass was removed before solvent extraction, metabolites associated with or retained within the mycelium may not have been recovered. Therefore, the crude extract used in the present study may not represent the complete spectrum of secondary metabolites produced by the fungal culture. Future studies combining extraction of both the culture filtrate and fungal biomass with comprehensive chemical characterization will be required to obtain a more complete metabolite profile. As a result, we could not quantitatively separate and compare the individual insecticidal contributions of fungal metabolites and silver nanoparticle cores to the overall toxic effect of Des-AgNPs. Third, this study mainly focused on evaluating the insecticidal activity and environmental persistence of biosynthesized silver nanoparticles, while the synergistic toxic interaction between crude extract components and nanocarriers requires further systematic verification in follow-up experiments. Future research will carry out targeted component detection of crude extracts and set complete matching control groups to clarify the respective toxic effects of fungal metabolites and silver nanoparticles, so as to further reveal the detailed toxic action mechanism of the nano-formulation.

## 5. Conclusions

This study evaluated the insecticidal activity, residual efficacy and metabolomic responses induced by silver nanoparticles biosynthesized from *M*. *anisopliae* crude extract, using *S*. *invicta* as the insect model. Our findings highlight the promising biocidal potential of fungus-synthesized silver nanoparticles for pest management. Characterization results confirmed that Des-AgNPs have a face-centered cubic structure and are spherical or nearly spherical, with good crystallinity and dispersion stability. In vitro toxicity assays indicated that Des-AgNPs exhibit strong toxicity against *S. Invicta* workers; field exposure tests demonstrated that Des-AgNPs retained insecticidal activity for longer than the crude-extract formulation under the tested conditions and a significantly longer residual efficacy period than the original biological formulation, indicating good stability. Metabolomics and KEGG enrichment analysis further revealed that Des-AgNPs treatment significantly disrupts multiple metabolic pathways in *S. invicta* workers, including glutathione metabolism, cytochrome P450-mediated detoxification, tyrosine/dopamine neurotransmitter metabolism, and ABC transporter-mediated efflux. These disruptions are indicative of oxidative stress, impaired energy metabolism, compromised membrane integrity, and disrupted neural transmission, which collectively contribute to the insecticidal effect of the nanoformulation. The combined metabolic alterations observed in the treated *S*. *invicta* workers may contribute to the overall physiological effects associated with Des-AgNPs exposure. This study confirms that Des-AgNPs, synthesized via green synthesis mediated by *M. anisopliae* crude extract, exhibit strong toxicity and stability against *S. invicta*. This finding offers the potential for a new, highly effective, stable, and environmentally friendly formulation for *S. invicta* control, and provides a theoretical basis for the development and application of novel AgNP-based pesticides. Importantly, this green biosynthesis approach provides a feasible strategy for developing biocidal nanomaterials, and further research with different fungal extracts and target pests is warranted.

## Figures and Tables

**Table 1:** Toxicity of five concentrations of Des-AgNPs against workers of *Solenopsis invicta* for 9 days.

Treatment	Concentration (mg/kg)	Regression Equation	Cumulative Mortality	Cumulative Corrected Mortality	LT_50_ (d)	Coefficient of Determination
Des-AgNPs	1000	y = −1.57 + 4.54x	100.00 ± 0.00 a	100.00 ± 0.00 a	2.286	0.958
	500	y = −1.7 + 3.46x	99.60 ± 0.89 a	99.56 ± 0.99 a	3.169	0.969
	250	y = −1.8 + 2.66x	82.00 ± 9.77 b	80.26 ± 10.71 b	4.77	0.977
	100	y = −1.76 + 2.15x	63.20 ± 8.44 c	59.65 ± 9.38 c	6.536	0.979
	50	y = −1.99 + 1.5x	34.00 ± 2.45 d	27.63 ± 2.69 d	-	0.988
CK	-	-	8.80 ± 1.79	-	-	-
AgNO_3_	-	-	10.67 ± 1.21	-	-	-

Note: Different lowercase letters within the same column indicate significant differences among different concentrations (*p* < 0.05).

**Table 2:** Toxicity stability of silver nanoparticles against *Solenopsis invicta* Workers under outdoor exposure for 9 days.

Treatment	Exposure Time (h)	Time–Mortality Regression Equation	Cumulative Mortality	Cumulative Corrected Mortality	LT_50_ (d)	Coefficient of Determination
Des-AgNPs	0	y = −1.57 + 4.54x	100.00 ± 0.00 a	100.00 ± 0.00 a	2.286	0.958
	24	y = −1.51 + 3.83x	100.00 ± 0.00 a	100.00 ± 0.00 a	2.519	0.954
	48	y = −1.68 + 3.71x	100.00 ± 0.00 a	100.00 ± 0.00 a	2.899	0.957
	72	y = −1.66 + 3.42x	100.00 ± 0.00 a	100.00 ± 0.00 a	3.116	0.971
	96	y = −1.79 + 3.36x	96.00 ± 0.00 a	95.00 ± 0.00 a	3.519	0.952
	120	y = −1.59 + 2.93x	89.20 ± 3.63 b	88.04 ± 4.02 b	3.496	0.998
*Metarhizium anisopliae* crude extract	0	y = −1.09 + 2.57x	98.80 ± 1.79 a	98.67 ± 1.98 a	2.683	0.989
	24	y = −1.06 + 2.21x	86.80 ± 4.38 b	85.39 ± 4.85 b	3.052	0.993
	48	y = −1.39 + 2.12x	72.40 ± 2.19 c	69.45 ± 2.43 c	4.587	0.952
	72	y = −1.37 + 1.33x	50.40 ± 5.27 d	45.09 ± 5.84 d	-	0.967
	96	y = −1.33 + 0.87x	33.60 ± 7.40 e	26.49 ± 8.20 e	-	0.970
	120	y = −1.3 + 0.85x	35.60 ± 8.17 e	28.71 ± 9.05 e	-	0.986
CK	0		9.67 ± 1.10		-	

Note: Different lowercase letters within the same column indicate significant differences among treatments (*p* < 0.05).

## Data Availability

The original contributions presented in this study are included in the article. Further inquiries can be directed to the corresponding author.

## Abbreviations

The following abbreviations are used in this manuscript:

UV-Vis	Ultraviolet–visible
TEM	Transmission electron microscopy
XRD	X-ray diffraction
DLS	Dynamic light scattering
UPLC-MS	Ultra-high-performance liquid chromatography–mass spectrometry
LT_50_	Median lethal time
LC_50_	5-day median lethal concentration
PCA	Principal component analysis
OPLS-DA	Orthogonal partial least squares discriminant analysis
ROS	Reactive oxygen species
